# New Genes in Traditional Seed Systems: Diffusion, Detectability and Persistence of Transgenes in a Maize Metapopulation

**DOI:** 10.1371/journal.pone.0046123

**Published:** 2012-10-03

**Authors:** Joost van Heerwaarden, Diego Ortega Del Vecchyo, Elena R. Alvarez-Buylla, Mauricio R. Bellon

**Affiliations:** 1 Biometris, Plant Sciences Group, Wageningen University and Research Center, Wageningen, The Netherlands; 2 Instituto de Ecología, Universidad Nacional Autónoma de México, México D.F., México; 3 Centro de Ciencias de la Complejidad, Universidad Nacional Autónoma de México, México D.F., México; 4 Bioversity International, Rome, Italy; 5 Interdepartmental Ph.D. Program in Bioinformatics, University of California Los Angeles, Los Angeles, California, United States of America; The Centre for Research and Technology, Hellas, Greece

## Abstract

Gene flow of transgenes into non-target populations is an important biosafety concern. The case of genetically modified (GM) maize in Mexico has been of particular interest because of the country’s status as center of origin and landrace diversity. In contrast to maize in the U.S. and Europe, Mexican landraces form part of an evolving metapopulation in which new genes are subject to evolutionary processes of drift, gene flow and selection. Although these processes are affected by seed management and particularly seed flow, there has been little study into the population genetics of transgenes under traditional seed management. Here, we combine recently compiled data on seed management practices with a spatially explicit population genetic model to evaluate the importance of seed flow as a determinant of the long-term fate of transgenes in traditional seed systems. Seed flow between farmers leads to a much wider diffusion of transgenes than expected by pollen movement alone, but a predominance of seed replacement over seed mixing lowers the probability of detection due to a relative lack of homogenization in spatial frequencies. We find that in spite of the spatial complexities of the modeled system, persistence probabilities under positive selection are estimated quite well by existing theory. Our results have important implications concerning the feasibility of long term transgene monitoring and control in traditional seed systems.

## Introduction

Since the introduction of genetically modified (GM) crops in the 1990s, gene flow into non-target populations has been a cause of concern [1]–[2]. The case of GM maize in Mexico has been of particular interest [3]–[4] because of the country’s status as center of origin and diversification of this important food crop [5]–[6]. In spite of a national moratorium on the planting of GM maize imposed in 1998, several studies have reported the presence of genetic elements of transgenic origin in populations of Mexican landraces [7]–[10].

Whereas biosafety issues such as introgression into wild relatives [11]–[13] and contamination of conventional food and seed supplies [14]–[15] were recognized early on, these reports have drawn attention to a hitherto unappreciated risk: gene flow into the traditional seed system that is typical of smallholder agriculture in the developing world. Unlike commercial farmers, most smallholders recover seed from the previous harvest, and frequently acquire seed from other farmers through a well-structured traditional system of rules, expectations and practices based on family and local social networks [16]–[19]. This traditional system effectively links all individual maize populations, or seed lots, into a single evolving metapopulation [20] in which the fate of an escaped transgene is subject to the long-term effects of population genetic processes such as drift, gene flow and selection.

All of these processes are determined to a large extent by seed management [21], the collection of farmer practices related to seed sourcing and use, including seed selection, planting, and acquisition [22] [17] [23].

Seed acquisition, denoted here as seed flow, is particularly important, as it strongly affects both drift and gene flow in the metapopulation [21]. Previous work has distinguished two forms of seed flow [23]: replacement (i.e. planting a field exclusively with seed obtained from another farmer) and mixing (i.e. planting a field with a mixture of seed saved from the farmer’s previous harvest and seed obtained from another farmer), which occur at different frequencies and have contrasting population genetic effects [21]. Replacement is the most common form of seed flow and mainly occurs in response to complete seed loss or when planting for the first time. Mixing is done in response to partial seed loss or as a form of experimentation [23]. Both mixing and replacement can involve seed from either inside or outside the farming community, although the former is much more common [24].

Although the importance of seed flow to the issue of transgene escape has been recognized [3] [10], research on agricultural biosafety in maize has been mostly limited to assessing the risk of cross-pollination between adjacent fields [25]–[26]. Empirical research is currently strongly restricted by biosafety regulations and can only address local and short-term consequences of transgene escape. Modeling studies therefore present an important alternative for exploring the issue of seed mediated gene flow and its implications by simulating future scenarios under contrasting assumptions and conditions on large spatial scales. So far there have been very few simulation studies addressing the effect of seed flow on the parameters relevant to biosafety policy (see [9]–[10] [27] for recent examples).

Seed flow may affect biosafety in a number of ways but here we focus on three aspects that we consider particularly important – namely diffusion, detectability and persistence of a transgene. Diffusion into non-target populations is at the heart of most biosafety concerns. Proposals for release of GM crops in Mexico have been aimed at release in regions of low maize diversity, implicitly assuming that long-distance seed dispersal can be ignored. Another key aspect for GM control is detectability. It has recently been shown that a very localized distribution of transgene frequencies lowers the probability of detection [9], prompting the need to evaluate the effects of seed flow on the spatial frequency distribution. Finally, persistence in response to natural selection is perhaps the most important long-term issue to be evaluated. Transgenes are designed to be agronomically superior in their target system, which, depending on the type of gene, may extend to traditional farming systems as well. While some of the currently available transgenic traits such as herbicide resistance may not be relevant to smallholder farmers if herbicides are not used, others may be, such as transgenes designed for biotic or abiotic stress resistance, like BT genes [3]. Such an advantage would become an evolutionary benefit within traditional seed systems. In classical population genetic models, positive selection increases the probability of a gene escaping extinction caused by genetic drift, leading to indefinite persistence and eventual fixation in the metapopulation. In classical models of undivided populations, this persistence (i.e. fixation) probability is determined by the initial gene frequency and the amount of genetic drift experienced by the metapopulation [28]. The extent to which crop metapopulations deviate from classical models in this respect remains to be established.

**Figure 1:**
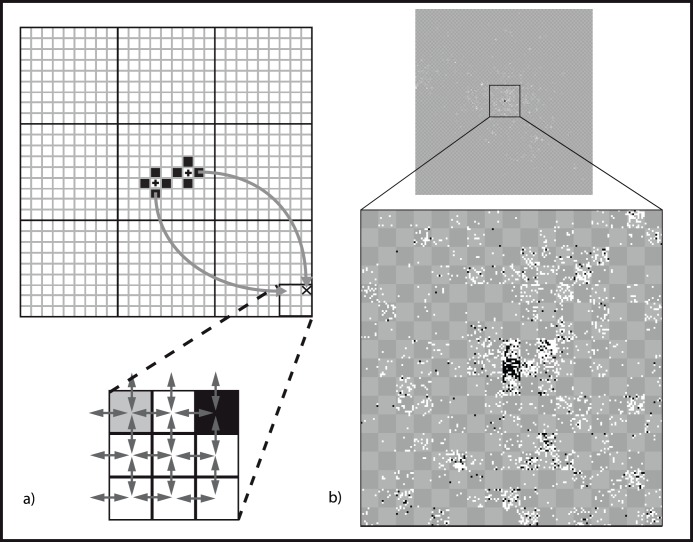
Diagram representing the metapopulation model (a) and example model output (b). The larger panel in (a) shows six villages with two positive farmers (black crosses) with seed flow indicated by gray arrows. Enlarged area shows a block of nine fields surrounding two fields that received contaminated seed. The field on the left mixed seed from a contaminated source, resulting in a frequency below that of the source field. The field on the right undergoes complete replacement and now has the same transgene frequency as the seed source. Panel (b) presents the spatial distribution of the transgene after 100 generations in the entire metapopulation (top) and in an enlarged area close to the focus of introduction (bottom). Transgene presence is marked in white and frequencies above 0.5 percent in black.

In this study, we provide a first example of using data on farmer-mediated seed flow to model the diffusion, detectability and persistence of a transgene. As an illustrative case study, we evaluate the long-term spatial dynamics of localized transgene introduction by modeling the recurrent growing of GM maize at a single location over 100 planting cycles, with the exception of the evaluation of persistence for which a one-time introduction is assumed. We use recently-compiled data [24] on traditional maize seed management in Mexico to parameterize a spatially-explicit, stochastic model describing the population genetics of a maize metapopulation subject to pollen and seed flow. This metapopulation consists of individual fields/seed lots cultivated within villages of which only a single central village is a cite of primary planting of GM maize (Figure 1). We consider only unconscious diffusion by excluding primary GM maize as a seed source, restricting the initial influx of transgenes into the metapopulation to be pollen mediated.

We evaluate the effect of substantial variations in seed flow parameters, with and without positive selection, by simulating five contrasting scenarios, one of which corresponds to the observed patterns of seed management in our sample of farmers and four that deviate from them in one or more seed flow parameters (Table 1). These scenarios assume constant pollen flow and combine three rates of seed mixing, two of seed replacement and two proportions of between-village seed flow, thereby providing a broad but manageable range of seed management conditions.

**Table 1:** Observed values for seed management data.

Parameter	Observed values
Number of ears used for planting (*F*)	290
Kernels per ear (*K)*	400
Probability of seed replacement (*ε)*	0.28
Probability of seed mixing (*μ*)	0.014
Proportion of seed used for mixing (*m*)	0.5
Probability of seed flow from non-local sources (*1 - V*)	0.14
Proportional contribution of pollen from a single neighbor to the gene pool of a field (*m_g_*)	0.015
Average number of pollen neighbors (ç)	1

We intend to demonstrate the value of explicit modeling of seed management when making predictions about the fate of escaped transgenes in landrace populations under proposed scenarios of introduction. We hope that our results will contribute to improved risk assessment and biomonitoring at centers of crop origin and in regions where traditional agriculture and diverse landraces dominate crop production systems.

## Results

### Diffusion

**Figure 2:**
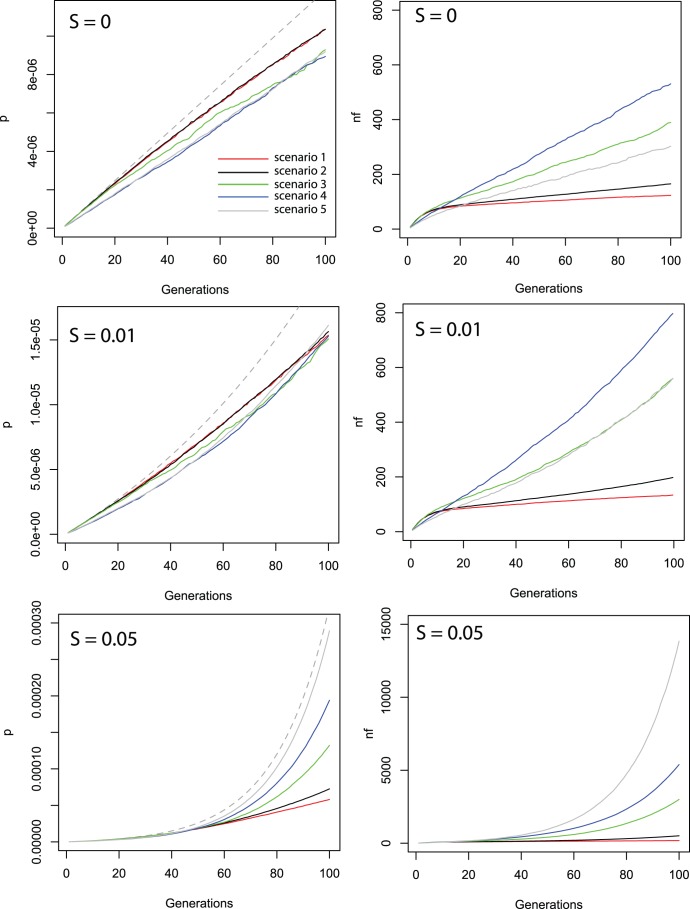
Accumulation of transgene frequency (p) and number of farmers with frequencies above 0.5 percent (nf) at s = 0 (top), s = 0.01 (middle) and s = 0.05 (bottom) for the five scenarios (red = 1,black = 2, green = 3, blue = 4, gray = 5). The gray dotted line shows expectations for p.

Recurrent planting of GM maize is expected to lead to the accumulation of transgenes in the metapopulation with a spatial distribution determined by the patterns of gene flow. In the absence of selection, the increase in transgene frequency is relatively insensitive to the specifics of seed flow (Figure 2). Under all five seed flow scenarios, gene frequency increases linearly at a rate somewhat inferior to the theoretical maximum expected for an infinite population undergoing recurrent influx of transgenic pollen (See methods). Under positive selection the effect of differences in seed flow parameters become visible, particularly for the highest selection coefficient of 0.05. Including seed flow leads to a faster increase in transgene frequency compared to the scenario without seed flow (scenario 1, Table 2). The introduction of seed mixing (scenario 2, Table 2) has a limited effect compared to the introduction of seed replacement (scenario 3–5, Table 2), probably due to the relatively low frequency of occurrence of the former. Increasing the frequency of introduction of seed from outside the village (scenario 4, Table 2) also leads to faster accumulation of transgenes, at the same total level of mixing and replacement. For the scenario with the highest frequencies of mixing, replacement and introduction of non-local seed (scenario 5, Table 2) the rate of accumulation approaches the theoretical maximum expected in an infinite population under constant influx and positive selection (See methods).

**Table 2:** Seed flow scenarios used in the simulations.

Scenarios	Pollen flow (*m_g_*)	Seed mixingprobability (*μ*)	Seed replacement probability (*ε)*	Probability of between-village seed flow (*1- V*)	Estimated N_e_
1	0.015	0	0.0	0.0	1,184,818,534
2	0.015	0.019	0.0	0.14	844,993,687
3	0.015	0.019	0.28	0.14	37,298,109
4	0.015	0.019	0.28	0.9999	37,298,109
5	0.015	0.28	0.28	0.9999	153,791,555

The number of affected farmers (i.e. whose fields contain more than 1% transgenic plants) responds more strongly to changes in seed flow parameters, regardless of the selection coefficient (Figure 2). In terms of both frequency and number of affected farmers, the difference between the zero seed flow scenario (1, Table 2) and the scenario with only seed mixing (scenario 2, Table 2) is small, however, again suggesting that at realistic rates seed replacement plays a more dominant role in transgene diffusion.

**Figure 3:**
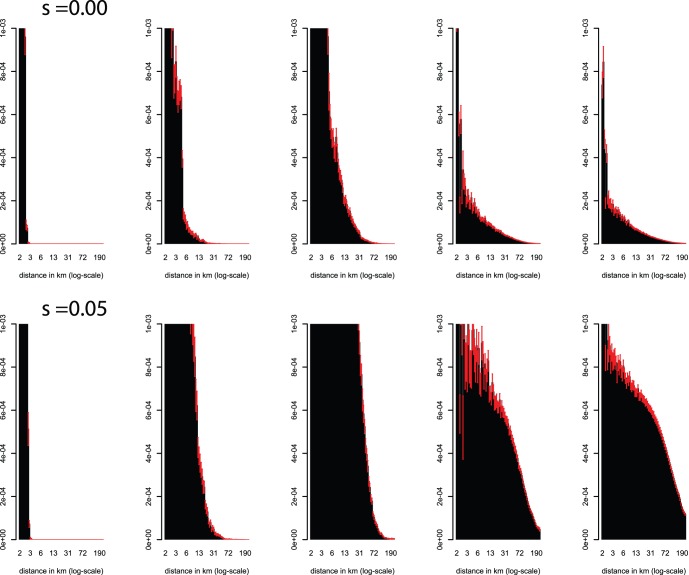
Frequency of the transgene vs. the distance from the focus of introduction (where the transgene is being introduced recurrently) at s = 0.00 (top) and s = 0.05 (bottom) for scenarios 1–5. Red whiskers indicate the standard error. Displayed values are truncated at p = 0.001.

As expected, seed mixing and replacement strongly affect the spatial distribution of transgenes (Figure 3). Whereas under the zero seed flow scenario (scenario 1, Table 2) transgene presence beyond three km from the focus of introduction after 100 years is negligible, the introduction of seed mixing and especially replacement leads to a much more even frequency distribution throughout the metapopulation, particularly when positive selection is strong. For scenario 3 (corresponding to realistic parameter values, Table 2) under the three selection coefficients (0, 0.01, 0.05), respective transgene frequencies of 2 10^−5^, 3 10^−5^ and 5 10^−4^, and fractions of affected farmers of 6 10^−4^, 1 10^−3^ and 3 10^−2^, are found as far as 50 km from the release site (Figure 3.). In terms of absolute numbers, more than ten million transgenic plants would grow at 50 or more kilometers from the point of introduction at the highest selection level, with more than 900 fields containing in excess of 1% transgenic plants.

### Detectability

Using a published biomonitoring scheme, consisting of 3 fields sampled for 50 random villages [29], we take repeated samples from the simulated metapopulation at 50 planting cycles after the cessation of 100 cycles of GM maize cultivation. We then measure the probability of transgene detection as the fraction of samples that contains at least one transgene. Detection results for the five seed flow scenarios without selection confirm earlier suggestions that seed flow may affect detectability of transgenes at a given frequency by increasing spatial homogeneity of transgene frequencies [9] (Table 3). As shown by the diffusion results above, in the absence of seed flow the spatial distribution of transgenes is highly skewed, meaning that a small number of fields close the source of introduction will contain high frequencies while those further away will have extremely low frequencies (Figure 3). This skewed distribution causes the observed detection probability to fall far below that expected based on conventional estimates that assume a homogenous population [29] (see methods). As seed mixing, replacement and between villages seed flow is introduced, the probability of detection also increases. However, only for the high seed flow scenario with 28 percent mixing does the detection rate begin to approach conventional expectations. Importantly, under realistic patterns of seed mixing (scenario 3, Table 2), the detection rate is only 0.14 instead of 0.74 as expected, reflecting the limited amount of homogenization associated with seed replacement compared to seed mixing.

**Table 3:** Detection probabilities of the transgene for each of the five scenarios.

Scenario	1	2	3	4	5
**Expected**	0.83	0.83	0.74	0.76	0.77
**Observed**	0.01	0.03	0.14	0.19	0.46

The observed probabilities refer to the average frequency of trials in which the positive transgene was detected among ten repetitions. Expected probabilities are simple binomial probabilities based on the assumption of spatially homogenous transgene frequencies (see methods).

### Persistence

Positive selection will inevitably lead to the long term persistence of a transgene unless random fluctuations in frequency, as a result of finite population size, are strong enough to cause the extinction of the gene. Starting from the low transgene frequency after a single cycle of planting of GM maize, the five scenarios and three selection levels show considerable variation in the probability of the transgene to persist indefinitely in the metapopulation (Table 4). Under a classical model of a single undivided population, such variation should reflect differences in metapopulation effective size (N_e_), a determinant of the amount of genetic drift [28]. Recent theory may be used to estimate N_e_ for the five scenarios [21] (see methods) and substituting these estimates into the classical model indeed leads to rather precise prediction of the persistence probabilities observed for our model.

**Table 4:** Persistence of the transgene, based on a starting frequency of 0.00001.

	selectioncoefficient	0.005		0.01		0.05	
Scenario	N_e_	Exp	Obs	Exp	Obs	Exp	Obs
**1**	11,848,115	0.947	0.923	0.997	0.989	1.000	1.000
**2**	8,450,219	0.876	0.854	0.985	0.980	1.000	1.000
**3**	374,069	0.088	0.123	0.169	0.257	0.604	0.843
**4**	374,069	0.088	0.085	0.169	0.180	0.604	0.732
**5**	1,538,842	0.317	0.276	0.533	0.461	0.978	0.947

Both the observed and expected persistence probabilities are shown for each of the six scenarios using two different selection coefficients. The observed probability of extinction in each scenario is estimated as the frequency of 100 simulations where the transgene frequency drops down to zero.

As expected, the highest estimated N_e_ is found in the absence of mixing and replacement (Scenario 1, Table 2), translating into probabilities of persistence close to 100 percent due to the low levels of genetic drift. Conversely, the scenarios with 28 percent seed replacement and 2 percent mixing (Scenarios 3 and 4, Table 2) have the lowest N_e_ and therefore relatively low probabilities of persistence. It is interesting that of the latter two scenarios the one corresponding to the observed seed management data (Scenario 3) deviates somewhat more from theoretical predictions than the scenario with higher rates of between-village seed flow (Scenario 4), in spite of having the same predicted N_e_. This points to a small but unexpected effect of the simulated village structure that cannot be accounted for by existing theory.

## Discussion

Predicting and managing the spread of transgenes beyond the target seed supply is of great importance for biosafety. Recent developments expanding the use of transgenes in crop plants to include the production of pharmaceuticals and other chemicals [30] have only increased the need for adequate evaluation of biosafety risks. Past experiences have shown that even within the commercial seed sector, preventing the intermixing of transgenic and conventional seed stock can be difficult [15]. The challenges of managing transgene flow in traditional seed systems in centers of crop origin are particularly daunting. Our study presents the first attempt to evaluate the potential importance of seed flow for the long-term fate of a transgene in a large crop metapopulation. By combining quantitative observations on traditional maize seed systems with a spatially explicit model, we are able to predict how deviations from observed practice may change the outcome of the introduction of a novel gene in terms of its diffusion, detectability and persistence. Our approach is a significant improvement over previous modeling studies that did not consider population genetics or seed management practices [31] [10].

As expected [3], seed flow is an important determinant of the population genetics of a transgenic element. Although in the absence of selection, seed mixing and replacement have a limited effect on the total frequency of the transgene, observed levels of replacement greatly affect the spatial distribution of the gene and the number of affected farmers. When selection is present, the frequency of mixing and replacement and the use of non-local seed sources notably influence the expected frequency of the transgene. Replacement, being the most common form of seed flow, thereby having the strongest effect.

Even though farmers predominantly rely on local seed sources, infrequent long-distance flow causes transgenes to spread much further than would be expected in the absence of seed flow. It is noteworthy that after 100 planting cycles, even under our relatively conservative scenario of 33 fields undergoing recurrent planting of GM maize, as many as 3% of farmers at 50 km from the original release sites are affected if the selection coefficient exceeds 0.05. This should be considered when evaluating biosafety of new releases based on distance from areas where landraces grow (see recent maps by CONABIO: http://www.biodiversidad.gob.mx/genes/proyectoMaices.html).

Although seed flow greatly increases the spatial diffusion of transgenes, the observed predominance of seed replacement over seed mixing translates into a limited rate of homogenization at the metapopulation level. In practical terms this means that true detection probabilities will be much lower than expected based on conventional calculations that assume a homogenous distribution [29]. In our case, only the scenario where seed mixing was equally frequent as replacement yielded a detection probability approaching theoretical expectations.

An important result from our simulations is that in spite of the complex spatial structure represented by our metapopulation, the probability of persistence of a transgene of known frequency and selective advantage is relatively well predicted by existing theory [28]. For the parameter ranges studied here, most of the variation in persistence probability at a given level of selection is explained by effective size as predicted from the model parameters using a recent mathematical crop metapopulation model [21]. The slight discrepancy observed between scenarios 3 and 4, in spite of their identical predicted population sizes, is the only instance where our model predictions deviate from theoretical expectations. Scenario 4 assumes unlimited seed flow between villages as in the mathematical model, while between-village seed flow is restricted in scenario 4. The fact that persistence in scenario 4 better fits theoretical predictions thus suggests that spatial restricted seed flow may cause an increase in effective size.

These results show that it is possible to make quantitative statements on the fate of transgenes, albeit under admittedly unrealistic assumptions of long-term persistence of the traditional maize production system. For example, given an approximate number of about 3 million small-scale maize farmers in Mexico [32], a rough theoretical estimate of the effective population size would be on the order of 100 million. This means that for a transgene with a selective advantage of 0.01, frequencies would have to remain below 10^−8^ to have a less than 5% chance of persistence. Detecting a single transgene at such frequencies with 95% probability would require testing around 750,000 maize ears under the assumption of homogeneity. Considering that true detection probabilities are much lower due to the uneven distribution of transgenes, it is unlikely that monitoring and controlling a selectively favored transgene would be feasible.

In all model predictions, selection is a key parameter. Although our simulated selection levels are within the range of published estimates for maize genes [33]–[34], we know very little about the potential fitness effects of different transgenes. Since transgenes are designed to be agronomically favorable and of large effect it is not unlikely that some are subject to strong positive selection, although others may turn out to be effectively neutral within traditional agro-ecosystems. Determining selective coefficients of particular transgenes under realistic conditions will thus be pivotal for designing sound detection and biosafety schemes and regulations and should be a research priority for an evidence-based biosafety system.

In this paper we have explored a limited and rather conservative example of transgene presence and diffusion to demonstrate the interaction between farmer practice and the population genetics of a new and rare genetic element. We have not tried to represent more extreme scenarios of transgene introduction such as may be occurring now or in the near future. Our analysis of the metapopulation dynamics of newly-introduced genes in traditional seed systems is by no means exhaustive. There are obviously many more scenarios and parameter combinations that may be explored, and there may be relevant aspects of seed management and that we have not included. Although the basic patterns of seed flow, such as localized exchange and predominance of seed replacement, will be similar for most traditional farming systems, it is likely that aspects of seed management vary by geography and environment [35] [24]. Some regions may for example have a tighter integration of traditional and modern seed systems than we have assumed [36], which would imply the occurrence of seed mixing between conventional and transgenic stock and faster transgene diffusion than predicted here. Nonetheless, we hope to have shown that knowledge on seed management is of great relevance for predicting the possible outcome of proposed scenarios of transgene release and is essential for establishing a rigorous and quantitative framework for biosafety policies.

## Materials and Methods

### Model Description

Our computational model extends recent population genetic models of traditional seed systems [9] [21] by providing spatially explicit representation of villages as well as long distance seed dispersal with a user-determined dispersal kernel. We simulate the temporal dynamics of a single, bi-allelic locus in a square grid of *n* fields assigned to *k* villages (Figure 1) where neighboring fields are connected by pollen and seed flow that can take place both within and between villages. Fields subject to primary planting of GM maize are assigned a starting frequency, *p = *0.5, of the transgenic allele. Recurrent planting of GM maize, such as assumed for the evaluations of diffusion and detectability, is modeled by resetting their frequency to 0.5 in each generation. The frequency change for each field in each generation is then modeled in four consecutive stages, where in each stage the starting frequency is designated by *p* and the new frequency by p*:

1. Pollen flow

Pollen flow is assumed to be deterministic. Fields receive a proportion *m_g_* of its genes from their η neighbors. The number of neighbors may be trimmed for the sake of realism. Fields at the corners and borders of the grid have two and three neighbors respectively. For a single field, transgene frequency after pollination is given by:
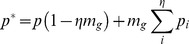
 with η ≤ 4.

1. Selection

Selection is also modeled deterministically under the assumption of complete dominance. Gene frequency after selection becomes:
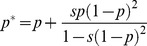

1. Drift

Each field is planted with *F* ears bearing *K* kernels, totaling *N* = *FK* diploid plants. We assume random mating within fields and *F*<<N so that the genetic sampling process is appropriately described as taking male and female gametes with replacement. Gene frequency in the paternal contribution to the next generation is given by:
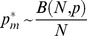



Whereas the frequency in the female contribution is:
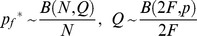



The double sampling describes the generation of *N* haploid gametes from *F* diploid ears. The post-drift gene frequency for each field is simply:
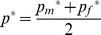

1. Seed flow

Seed flow occurs by mixing and replacement, respectively. For both types of seed flow there is a single seed source for each receiving field in each generation. Replacement occurs with a probability *ε* and involves replacement of the entire seed lot with that of another farmer. Transgenic frequency after replacement is given by 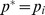. Where *p_i_* is the frequency in the source field. Mixing takes place with a probability *μ* and involves supplementing a fraction *m* of the native seed lot with seed from a another farmer. Frequency after mixing thus becomes: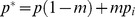. Seed sources are chosen randomly from non-replaced seed lots from within or from outside the village with probabilities *V* and (1- *V*) respectively. Each source outside the village is defined by a random angle δ from a uniform distribution and a random distance *d* (according to a chosen dispersal kernel). If a replaced or primary GM maize seed lot is chosen or if the coordinates fall within the same village or outside the grid, a new random source is selected. The exclusion of GM maize seed lots as seed sources, imposed to model unconscious diffusion, can obviously be lifted if so desired. Source code of our model is available upon request.

### Information on Seed Systems and Parameterization

#### Seed management data

To obtain estimates for seed related model parameters we used raw data from a recently published socioeconomic survey [24] that was carried out in 2003 with the heads (i.e. the person making decisions on domestic and farm issues) of 400 randomly-selected maize-producing households at 20 sites (20 households per site) randomly selected among 46 municipalities comprised in four transects across an altitudinal gradient from 10 to 2980 masl in five states in Central Mexico.

All maize farmers interviewed relied on traditional seed systems and planted mostly landraces, although some of them had experimented with improved varieties as well. In addition to socio-economic data, this survey contained questions specific to maize seed management such as quantities, geographic origins and history of the seed planted. Estimates of pollen flow were taken from the literature [26]. Because most parameters differed substantially between maize-growing environments and in order to represent a single environment, only data for the highland environment was used, with the exception of seed source distances, which were obtained from the full dataset to assure sufficient sample size. The parameter estimates derived from these data are presented in Table 1. The reported distances of seed sources confirmed a prevalence of local seed flow with rare instances of long-distance seed flow, as reported in other studies. We fit a lognormal distribution to observed distances using the *fitdistr* routine contained in the MASS package of the R statistical software [37]. Seed flow distances between villages in our model were set to correspond to real between-village distances by scaling the average between-village distance in our simulations to match the average neighbor distance in a Delaunay triangulation of the coordinates of 911 mapped highland villages (>300 inhabitants) in a rectangular section of the study region (information: INEGI, http://www.inegi.org.mx).

### Parameterization and Model Scenarios

Our simulated metapopulation contained one million fields divided equally over 10,000 villages, with a single village in the center containing 33 fields planted with GM maize. We explored five different scenarios of seed flow (Table 1), covering a wide range of seed management types ranging from the complete absence of seed flow to a high seed flow scenario of 28 percent seed replacement and mixing and a 99.99 percent probability of between-village flow. Scenario number three represented the observed seed management parameters as estimated from our survey data, i.e. infrequent seed mixing, frequent seed replacement and predominantly local seed flow. For the evaluation of transgene diffusion, continuous planting by the same 33 farmers was simulated for 100 generations and the results averaged over 100 replications. We tested selection coefficients of 0, 0.01 and 0.05. The expected maximum rate of transgene frequency increase in an undivided population of infinite size is simply the deterministic increase caused by pollen flow from positive fields and positive selection, represented by: 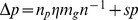



For the detectability evaluation the five scenarios were simulated in the absence of selection (s = 0), for 150 generations with planting of GM maize by 33 farmers ceased after 100 generations. For each replication, 10,000 samples were simulated, consisting of 50 randomly-selected villages, three fields per village, seven ears per field and 165 seeds per ear. This sampling scheme was designed to be quantitatively similar to Ortiz-Garcia *et al.* [29] in their 2003–2004 biomonitoring survey of Mexican landraces. Detection was modeled as a set of Bernoulli trials, in which each sampled field has a probability of detection of 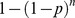, where 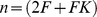 [9], and *p* is the transgene frequency in that field. The expected overall detection probability under the assumption of homogeneous frequencies is simply given by the above formula with *F* and K replaced by the total number of sampled ears and kernels respectively and by replacing *p* by the mean transgene frequency in the metapopulation [29].

Theoretical expectations for persistence are represented by 
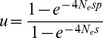
 [28], where *u* is the persistence (or fixation) probability and N_e_ is the variance effective population size. Given p and s, higher values of N_e_ lead to higher persistence probabilities because of a lower chance of losing the new gene due to drift. N_e_ was calculated as the long-term N_e_ derived from the mean coalescent time T (N_e_ = 2T), given by the crop metapopulation model by van Heerwaarden et al. 2010 [21], with model parameters set as in Table 1.

For the evaluation of persistence a smaller metapopulation of only 100 villages was simulated to limit computational time. In this case 1000 replications were run of a single planting cycle of GM maize by 33 farmers, leading to a starting frequency of p = 1.24×10^−5^. Selection was set to 0.005, 0.01 and 0.05. Simulations were run until transgene frequency reached either 0 (extinction) or 0.001 (persistence). The threshold value of 0.001 for persistence was used to limit computational time and based on the fact that for this frequency the probability of fixation approaches unity under realistic values of N_e_.
